# Recompression after percutaneous transforaminal endoscopic decompression for degenerative lumbar spinal stenosis: risk factors and outcomes of two different reoperation procedures

**DOI:** 10.3389/fsurg.2024.1392215

**Published:** 2024-06-24

**Authors:** Shuo Yuan, Aobo Wang, Ning Fan, Peng Du, Tianyi Wang, Jian Li, Wenyi Zhu, Lei Zang

**Affiliations:** Beijing Chaoyang Hospital, Capital Medical University, Beijing, China

**Keywords:** lumbar spinal stenosis, pain, postoperative, risk factor, treatment outcome, minimally invasive, geriatric patients

## Abstract

**Purpose:**

To determine the risk factors for recompression after percutaneous transforaminal endoscopic decompression (PTED) for the treatment of degenerative lumbar spinal stenosis (DLSS) and compare the outcomes of PTED and posterior lumbar interbody fusion (PLIF) as revision surgery.

**Methods:**

We retrospectively evaluated 820 consecutive DLSS patients who underwent PTED at our institution. 26 patients developed postoperative recompression and underwent reoperation. In total, 208 patients with satisfactory clinical outcomes were enrolled in the control group. The demographic and imaging data of each patient were recorded. Univariate and multivariate analyses were performed to assess risk factors for recompression. Additionally, patients with recompression were divided into PTED and PLIF groups according to the reoperation procedure. The clinical outcomes of the two groups were compared using independent-sample t-tests.

**Results:**

The grade of surgical-level disc degeneration [odds ratio (OR): 2.551, *p* = 0.045] and the number of disc degeneration levels (OR: 11.985, *p* < 0.001) were independent risk factors for recompression after PTED. There was no significant difference in the visual analog score (VAS) and Oswestry disability index (ODI) two weeks postoperatively between the PTED and PLIF groups for surgical treatment. However, the mean VAS of back pain (14.1 vs. 20.5, *p* = 0.016) and ODI (16.0 vs. 21.8, *p* = 0.016) of patients in the PLIF group were smaller than those in the PTED group at the final follow-up.

**Conclusion:**

More severe degeneration and degenerated levels indicate a higher recompression rate after PTED. Although both PTED and PLIF could achieve immediate relief postoperatively in the treatment of recompression, the final follow-up results showed that the outcome of PLIF appeared better than that of PTED.

## Introduction

Percutaneous endoscopic lumbar discectomy (PELD) has been widely used in the treatment of lumbar disc herniation (LDH) (1, 2). With the development of surgical instruments and techniques, percutaneous transforaminal endoscopic decompression (PTED) has been increasingly considered a surgical option for lumbar foraminal, lateral recess, or central canal stenosis. PTED can remove the herniated disc and the osteophytes, hypertrophic ligament flavum, and hyperplastic articular process. Studies have shown that PTED is safe and effective, with the advantages of less trauma, faster rehabilitation, and less adjacent segment degeneration compared to traditional open surgeries (3–5). With the widespread use of this surgical technique, surgical complications, and clinical outcomes of PTED have gained more attention. Studies showed that 3.5%–17.7% of patients with degenerative lumbar spinal stenosis (DLSS) require reoperation after minimally invasive surgeries (6–8).

As one of the leading causes of reoperations, recurrent LDH (rLDH), defined as disc herniation that recurs at the same level after the primary surgery, has been investigated in many previous studies (9). A meta-analysis showed that rLDH usually occurred within 6 months after PTED, with a prevalence of 3.6% (10). The risk factors for rLDH include obesity, age, disc height index, Modic changes, sagittal range of motion, and adjacent segment degeneration (11–13). However, the all-cause recompression of the spinal canal or nerve root after surgical treatment of DLSS, which accounts for 52.1%–100% of reoperation cases (14), has not been fully investigated. Haimoto (15) reported that a decrease in disc height or progression of disc wedging was a risk factor for recompression after microscopic decompression; however, the sample size was relatively small (7 in the recompression group). To the best of our knowledge, few studies have focused on the risk factors for recompression after PTED.

The selection of procedures as revision operations is another noteworthy problem. Regarding rLDH, both PELD and traditional open surgery can achieve satisfactory outcomes (9, 16–18). However, the causes of nerve root compression are more complex in patients with DLSS than those with only LDH, including herniated disc, residual ligament flavum, thickening of the facet joints, and scarring (14). Therefore, the treatment of recompression is more complicated. Although many studies have considered spinal fusion the optimal revision choice (19–22), some clinicians support minimally invasive and non-fusion concepts to reduce surgical trauma (14, 23), especially for those without lumbar instability. However, whether PTED can achieve better efficacy than traditional spinal fusion has not been determined.

In a previous study, we have reported the incidence and risk factors of early readmission and reoperation after PTED in treating DLSS (24). In this study, we further analyzed the previous collected patient data and focused on recompression, which is a middle-to-long-term complication after PTED. We retrospectively reviewed 26 patients with recompression after PTED and analyzed their clinical and radiological data compared to control patients to explore the risk factors for recompression after PTED. In addition, patients with recompression were grouped according to the surgical procedure [PTED or posterior lumbar interbody fusion (PLIF)]. The postoperative outcomes were compared between the two groups to optimize the surgical choice. We hypothesized that imaging characteristics might be risk factors for recompression after PTED. Furthermore, because of these imaging characteristics, the postoperative efficacy of PTED for the treatment of recompression may not be as good as that of PLIF.

## Material and methods

### Patient selection

This study retrospectively evaluated 820 consecutive patients with a lateral recess or foraminal stenosis who underwent unilateral and single-level PTED at our institution between January 2016 and March 2021. The inclusion criteria for patients with DLSS were: (1) unilateral lateral recess or foraminal stenosis diagnosed based on clinical symptoms, physical examination, and imaging; (2) a sudden onset but the symptoms seriously affected work and life, or symptoms with no relief after at least 3 months of conservative treatment. The exclusion criteria were: (1) symptoms caused only by LDH; (2) instability or more than grade I spondylolisthesis at the responsible segment; (3) bilateral PTED or more than one surgical level; (4) follow-up for less than 12 months; (5) history of lumbar surgery; (6) insufficient clinical or imaging data; and (7) concomitant conditions affecting the lumbar spine, including fractures, tuberculosis, and tumors.

Among 820 patients, 26 developed recompressions postoperatively and underwent reoperation. This study defined recompression as LSS that recurred on the same side and at the same level after PTED, with a minimum one-month pain-free interval. The inclusion criteria for patients with recompression were: (1) LSS diagnosed based on clinical symptoms, physical examination, and imaging; (2) a sudden onset, but the symptoms seriously affected work and life, or symptoms with no relief after at least 3 months of conservative treatment. The exclusion criterion was short-term complications after the initial operation, such as insufficient decompression. The characteristics of the patients with recompression are listed in Table 1. The average time of reoperation is 17.3 months. A control group was matched according to the surgery date. In order to ensure a relatively adequate sample size, the matching ratio was 1:8 to identify the risk factors for recompression. A total of 208 patients in the control group did not have any surgical complications and were assessed as having excellent or good clinical outcomes according to the modified MacNab criteria at the final follow-up (March 2022). This study was performed in accordance with the Declaration of Helsinki. Approval for the study was obtained from the institutional ethics committee.

**Table 1:** Characteristics of the patients with recompression.

No.	Age (years)	Gender	Surgical level	Reoperation	Time of reoperation (months)	Follow-up duration (months)
1	47	Male	L4/5	PTED	2	68
2	53	Male	L4/5	PTED	2	58
3	83	Male	L4/5	PTED	9	48
4	51	Male	L4/5	PTED	2	47
5	55	Male	L5/S1	PLIF	4	45
6	87	Male	L4/5	PLIF	21	44
7	75	Male	L4/5	PLIF	3	41
8	69	Female	L4/5	PLIF	2	40
9	87	Male	L4/5	PTED	25	37
10	57	Female	L5/S1	PTED	29	30
11	73	Male	L4/5	PTED	42	27
12	85	Female	L4/5	PTED	6	27
13	81	Male	L4/5	PTED	30	24
14	55	Female	L4/5	PLIF	56	16
15	69	Female	L4/5	PTED	14	15
16	70	Male	L4/5	PTED	40	15
17	78	Female	L4/5	PTED	2	14
18	65	Male	L4/5	PLIF	21	13
19	69	Male	L4/5	PTED	10	13
20	74	Female	L4/5	PLIF	48	13
21	68	Male	L4/5	PLIF	5	12
22	66	Male	L4/5	PLIF	4	12
23	59	Female	L5/S1	PLIF	51	12
24	63	Male	L5/S1	PLIF	5	12
25	56	Female	L4/5	PTED	11	2
26*	48	Female	L4/5	PTED	5	27

* The recompression in this patient was treated at another institution.

PTED, percutaneous transforaminal endoscopic decompression; PLIF, posterior lumbar interbody fusion.

### Surgical methods

#### PTED

**Figure 1:**
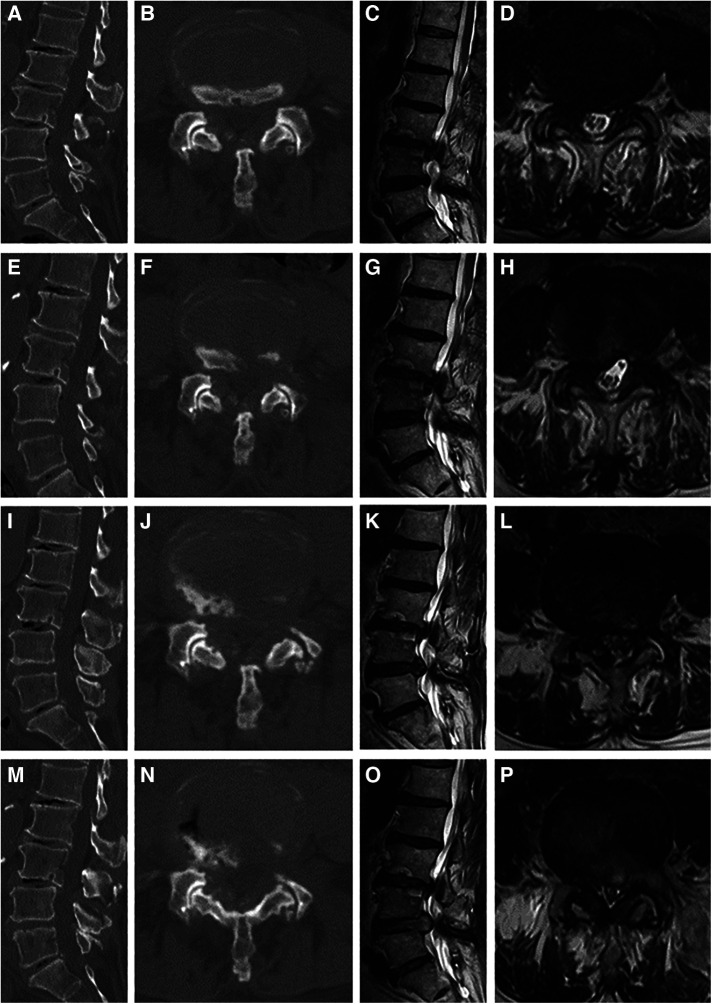
Case illustration of percutaneous transforaminal endoscopic decompression (PTED) in the treatment of recompression. (**A**–**D**) Computed tomography (CT) and magnetic resonance (MR) images before the primary PTED showed left-sided lateral recess and foraminal stenosis at the L4/5 level. (**E**–**H**) Postoperative images showed adequate decompression. (**I**–**L**) CT and MR images 10 months postoperatively showed that recompression occurred due to disc herniation and osteophytes. (**M**–**P**) Post-reoperative images.

The primary and revision PTED procedures were performed by a senior surgeon with an experience of more than 100 PELD procedures. In patients with multilevel radiographic stenosis, local blocking was performed to identify the level of responsibility. The details of PTED procedure were described in the previous study (24). Figure 1 is a case illustration of PTED in the treatment of recompression.

#### PLIF

**Figure 2:**
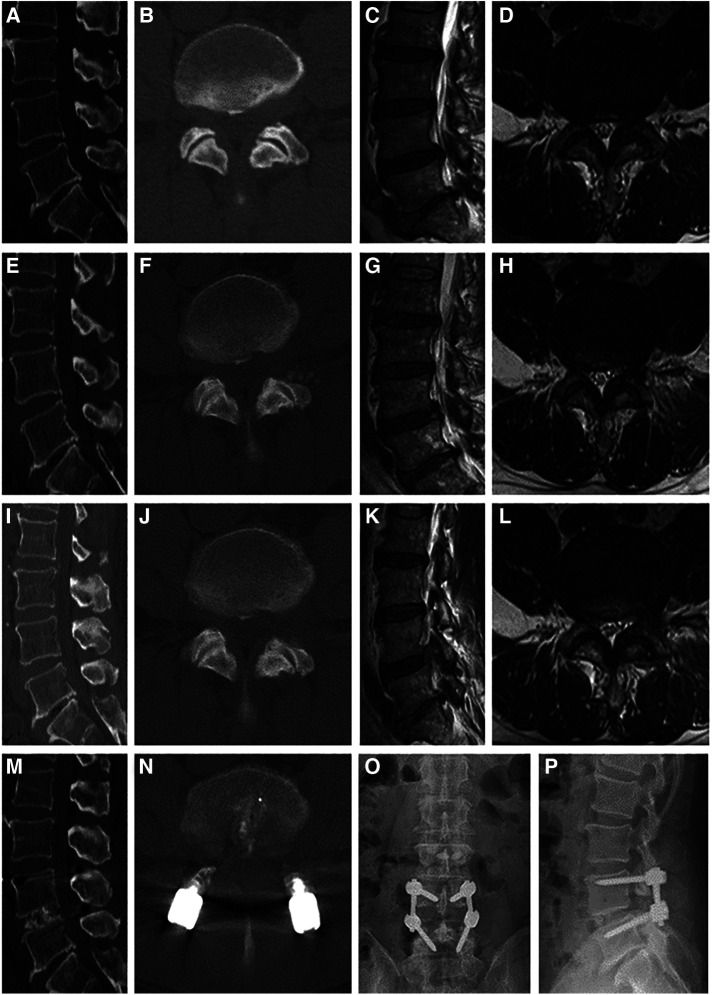
Case illustration of posterior lumbar interbody fusion in the treatment of recompression. (**A**–**D**) Computed tomography (CT), and magnetic resonance (MR) images before the primary PTED showed left-sided lateral recess and foraminal stenosis at the L4/5 level. (**E**–**H**) Postoperative images showed adequate decompression. (**I**–**L**) The patient reported recurrent pain months postoperatively, and CT and MR images confirmed recompression. (**M**–**P**) Post-reoperative x-ray and CT images demonstrated that the pedicle screws were in a good position.

The patient was placed in the prone position after general anesthesia. The surgical level was determined based on the C-arm image, and a posterior midline incision was made to expose the surgical segment. Two pedicle screws were inserted in the superior and inferior vertebrae. A hemilaminectomy was performed to expose the spinal dura mater. Facetectomy was performed on the symptomatic side to expose the nerve root and intervertebral disc. Subsequently, the disc was thoroughly removed, and the cartilage endplate was scraped. Autologous bone was then implanted into the disc space, and an appropriately sized cage packed with bone autograft was inserted. The pedicle screws were connected to screw rods and fixed with nuts. The incision was closed after the irrigation and drainage tube placement (Figure 2).

### Data collection and assessment

Demographic and radiological parameters of all enrolled patients before the primary PTED were collected. Demographic parameters included age, gender, body mass index (BMI), smoking and drinking status, comorbidities, and surgical level. Radiological parameters such as grade of surgical-level disc degeneration, number of degenerative lumbar discs, grade of lumbar spinal stenosis, degenerative lumbar scoliosis, lumbar lordosis, disc wedge, Modic changes (MCs), and disc height index (DHI) were measured and compared between the case and control groups. Two trained orthopedic surgeons performed all measurements using DICOM (version 3.1) viewer software (Neusoft PACS/RIS), and the mean of the measurements was calculated.

Degenerative lumbar scoliosis was defined as a Cobb angle >10°. Lumbar lordosis is defined as the angle between the superior endplates of L1 and S1. DHI was calculated as: [(anterior disc height + posterior disc height)/(superior disc depth + inferior disc depth)] × 100. The disc wedge was defined as the angle between the lower endplate of the upper vertebra and the upper endplate of the lower vertebra. Scoliosis, lumbar lordosis, DHI, and disc wedge measurements were performed on x-ray images. Based on magnetic resonance imaging (MRI), disc degeneration at the surgical level was graded according to Pfrrmann (25), and the number of degenerative discs was defined as the levels with disc degeneration of grade IV or higher. MCs were defined in previous studies (26). Lumbar spinal stenosis was graded according to Schizas criteria (27).

### Assessment of surgical effects

Among the patients with recompression, 1 underwent reoperation at another institution, and 1 underwent PTED in Jan 2022 (a follow-up duration less than 12 months). These 2 patients were included in the analyses of risk factors but were not included in comparing clinical outcomes. Therefore, 24 patients were enrolled in this study. They were divided into PTED (*n* = 13) and PLIF (*n* = 11) groups.

We assessed the clinical symptoms and outcomes of the recompression patients by reviewing medical records, outpatients, and telephone follow-ups. The visual analog pain score (VAS) and Oswestry disability index (ODI) were scored before the primary operation, 2 weeks and 3 months after the primary operation, before the reoperation, 2 weeks, 3 months, and 1 year after the reoperation, and at the final follow-up. For patients who underwent revision surgery within three months after the initial PTED, the pain scores before the second surgery were considered as their scores at 3 months after the primary operation, since we believe that the symptoms of these patients were difficult to alleviate through non-surgical treatments. The modified MacNab criteria were also used to evaluate clinical outcomes. Excellent or good outcomes were considered to be satisfactory. In addition, the complications of reoperation were collected.

### Statistical analysis

Data were analyzed using statistical software (SPSS version 23.0 for Windows, IBM) and R 4.1.3. Continuous variables are presented as mean ± standard deviation. In this study, we adopt a primary component analysis (PCA)-based cluster analysis as an exploratory method to determine the appropriateness of subgrouping. Univariate analyses included independent samples t-tests and Pearson's chi-square tests. Logistic regression analysis was performed to identify independent risk factors. Based on the results from the regression analysis, a nomogram for recompression probability was constructed, and the performance of the nomogram was assessed using the area under the receiver operating characteristic curve and a visual calibration plot. Paired sample t-tests were used for preoperative and postoperative comparisons. Independent sample t-tests were used to compare the clinical outcomes between the PTED and PLIF groups. Statistical significance was set at *p* < 0.05.

## Results

**Figure 3:**
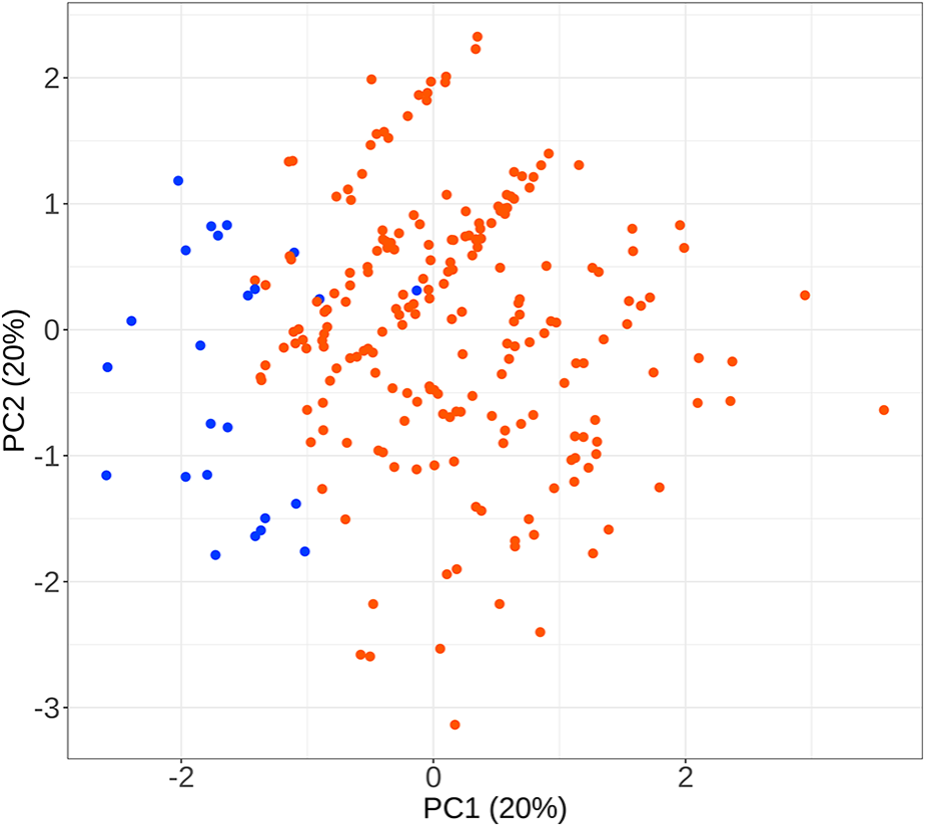
The results of primary components analysis of demographic parameters in recompression and control groups. The result showed that patients in the recompression (blue) and control (red) groups could be divided almost definitely by 2 primary components.

**Figure 4:**
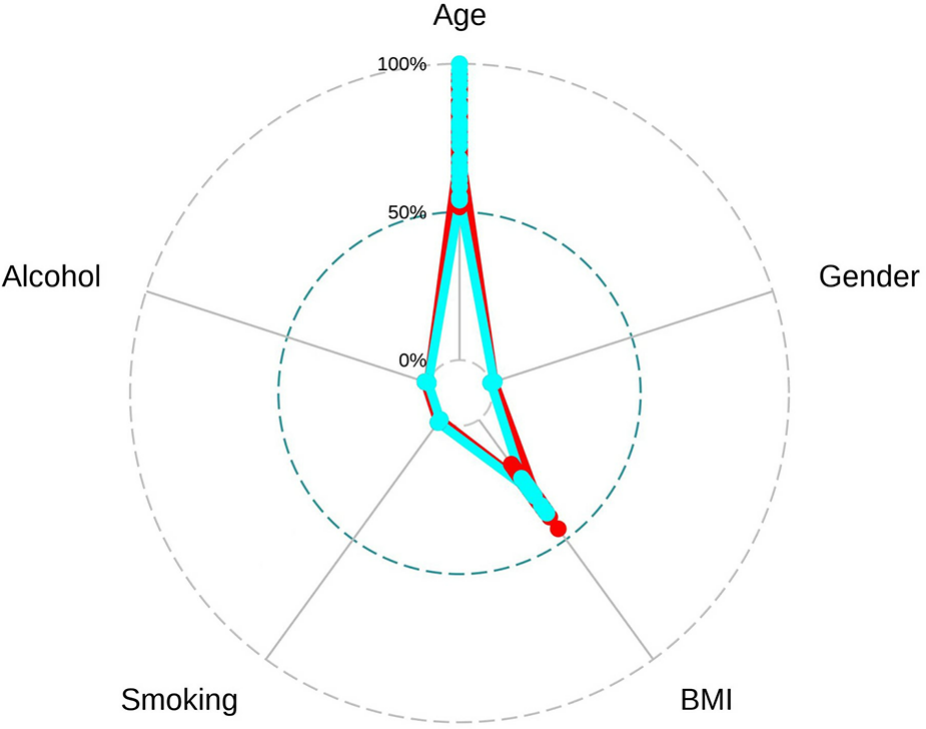
The radar plot revealed that the 2 primary components tend to be age and body mass index (BMI). Relatively significant differences were found referring to the 2 variables between recompression (blue) and control (red) groups.

**Figure 5:**
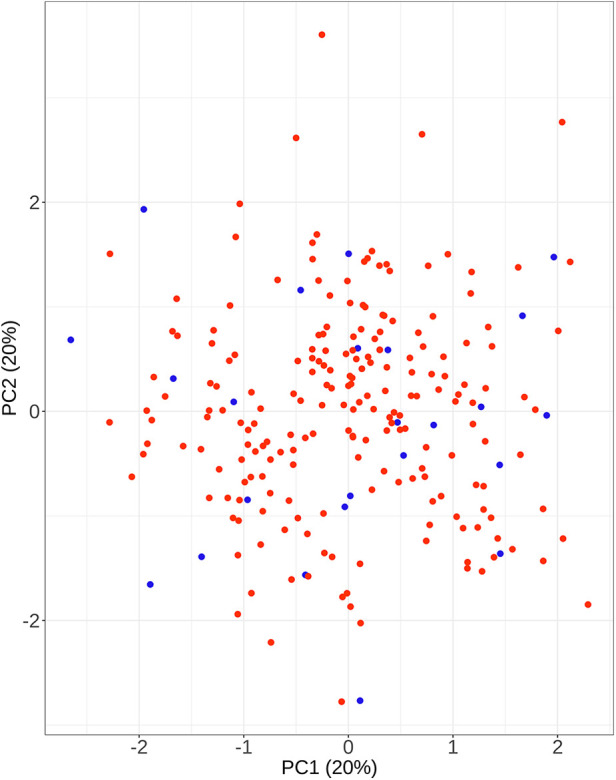
The results of primary components analysis of clinical parameters in recompression and control groups. The result showed that patients in the recompression (blue) and control (red) groups could not be divided appropriately.

**Figure 6:**
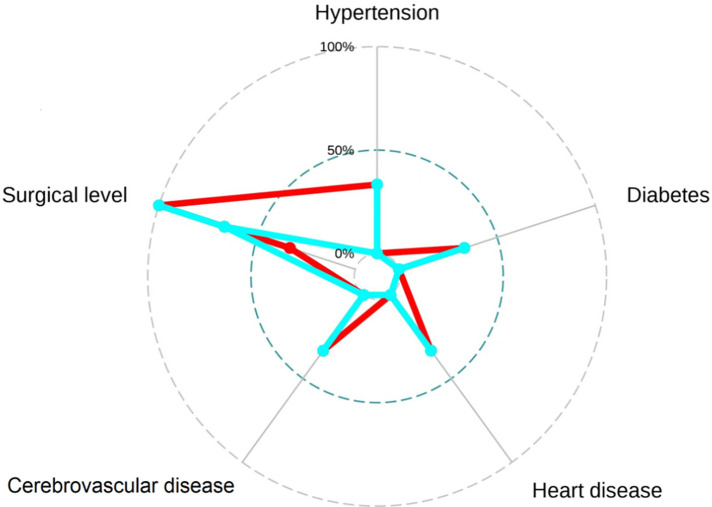
The radar plot revealed no significant differences were found between recompression (blue) and control (red) groups.

**Figure 7:**
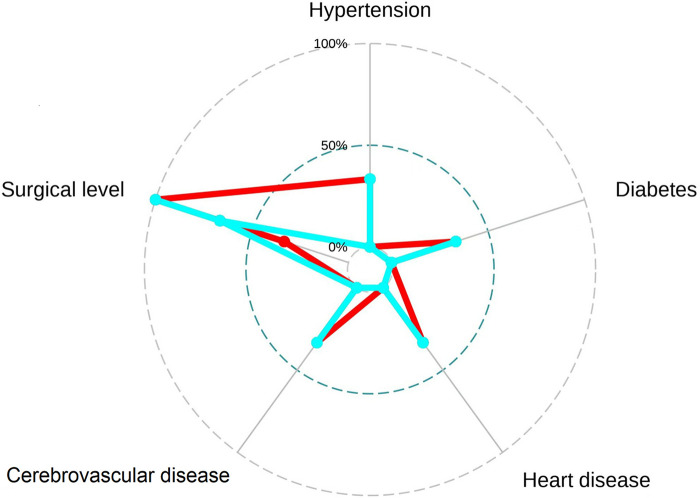
The results of primary components analysis of radiologic parameters in recompression and control groups. The result showed that patients in the recompression (blue) and control (red) groups could be divided almost definitely by 2 primary components.

**Figure 8:**
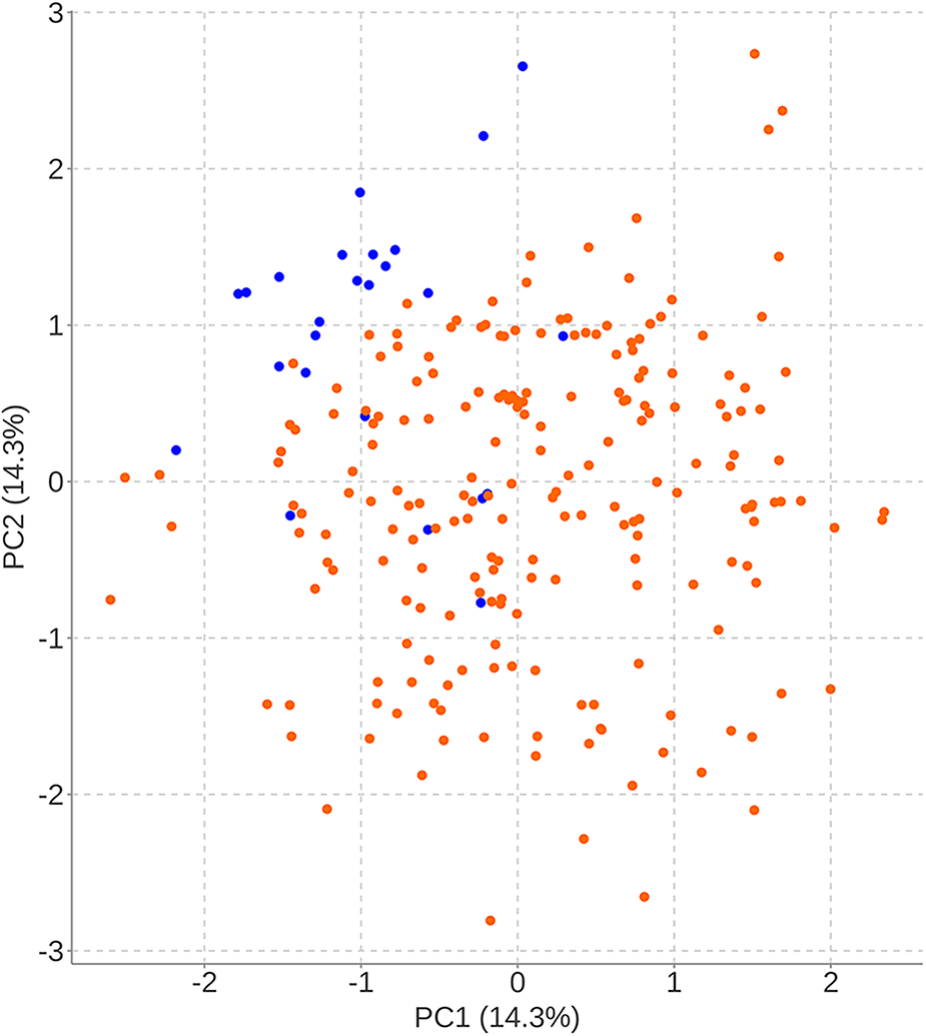
The radar plot revealed that the 2 primary components tend to be the grade of surgical-level disc degeneration and the number of degenerative lumbar discs. Relatively significant differences were found referring to the 2 variables between recompression (blue) and control (red) groups.

To facilitate the comparison of the recompression and control groups, we categorized 5 demographic parameters, 5 clinical data, and 8 radiological parameters and performed a PCA-based cluster analysis. Patients in the recompression and control groups could be divided into two groups based on two primary components of demographic parameters and two radiological parameters but not clinical data (Figures 3–8). The primary components are revealed in the radar plot. There tend to be grades of surgical-level disc degeneration and the number of degenerative lumbar discs in radiological parameters, age, and BMI in demographic parameters. Relatively significant differences were found in the four variables between the recompression and control groups using radar plots. Univariate analyses and multivariate logistic regression were conducted to verify the conclusions of PCA.

The results of the univariate analysis of the demographic characteristics are shown in Table 2. The mean age of patients with recompression was significantly higher than that of control patients (67.0 vs. 62.1, *p* = 0.021). There were no significant differences in gender (*p* = 0.411), BMI (*p* = 0.719), smoking status (*p* = 1.000), drinking status (*p* = 1.000), comorbidities (*p* > 0.05), or surgical level (*p* = 0.423) between the two groups. Regarding radiological parameters, the results of the univariate analysis showed that patients with recompression had higher grades of surgical-level degeneration (4.2 vs. 3.9, *P* = 0.007) and more degenerated levels (3.5 vs. 2.0, *P* < 0.001). The results are summarized in Table 3.

**Table 2:** Comparisons of demographic and clinical data between patients in recompression group and control group.

	Recompression group (*n* = 26)	Control group (*n* = 208)	*P* value
Age (years)	67.0 ± 12.1	62.1 ± 10.1	0.021*
Gender (Male/Female)	16/10	109/99	0.411
BMI (kg/m^2^)	25.8 ± 3.1	25.3 ± 3.6	0.719
Current smoker, *n* (%)	6 (23.1%)	54 (26.0%)	1.000
Alcohol drinking, *n* (%)	3 (11.5%)	25 (12.0%)	1.000
Comorbidities			
Hypertension. *n* (%)	16 (61.5%)	91 (43.8%)	0.098
Diabetes mellitus, *n* (%)	8 (30.8%)	60 (28.8%)	0.822
Heart disease, *n* (%)	7 (26.9%)	29 (13.9%)	0.090
Cerebrovascular disease, *n* (%)	6 (23.1%)	25 (12.0%)	0.127
Surgical level, *n* (%)			0.423
L2/L3, L3/L4	0 (0)	14 (6.7%)	
L4/L5	22 (84.6%)	151 (72.6%)	
L5/S1	4 (15.4%)	43 (20.7%)	

* Statistically significant at *p* < 0.05.

BMI, body mass index.

**Table 3:** Comparison of imaging parameters between patients in the recompression and control groups.

	Recompression group (*n* = 26)	Control group (*n* = 208)	*P* value
Number of disc degeneration levels	3.5 ± 0.9	2.0 ± 0.8	<0.001*
Grade of surgical-level disc degeneration	4.2 ± 0.5	3.9 ± 0.7	0.007*
Modic changes, *n* (%)	8 (30.8%)	50 (24.0%)	0.473
Degenerative lumbar scoliosis, *n* (%)	5 (19.2%)	27 (13.0%)	0.369
Lumbar lordosis, (°)	36.0 ± 14.9	39.0 ± 13.9	0.296
Disc wedge (°)	7.8 ± 2.8	8.8 ± 4.5	0.110
Disc height index	24.0 ± 6.0	26.2 ± 5.2	0.050
Schizas grade, *n* (%)			0.687
A	14 (53.9%)	100 (48.1%)	
B	4 (15.4%)	21 (10.1%)	
C	6 (23.1%)	65 (31.3%)	
D	2 (7.7%)	22 (10.6%)	

* Statistically significant at *p* < 0.05.

Multivariate logistic regression analysis demonstrated that the grade of surgical level degeneration [odds ratio (OR) = 2.551, 95% confidence interval (CI): 1.021–6.371, *p* = 0.045] and the number of levels with disc degeneration (OR = 11.985, 95% CI: 4.432–32.412, *p* < 0.001) were independent risk factors for recompression (Table 4). Patients with a higher grade of surgical-level disc degeneration and more levels of disc degeneration are more likely to develop recompression after surgery than those who do not.

**Table 4:** Multivariate regression model of the predictors for recompression.

	Significance	Odd ratio	95% confidence interval
Number of disc degeneration levels	<0.001*	11.985	4.432–32.412
Grade of surgical-level disc degeneration	0.045*	2.551	1.021–6.371
Age	0.259		

* Statistically significant at *p* < 0.05.

**Figure 9:**
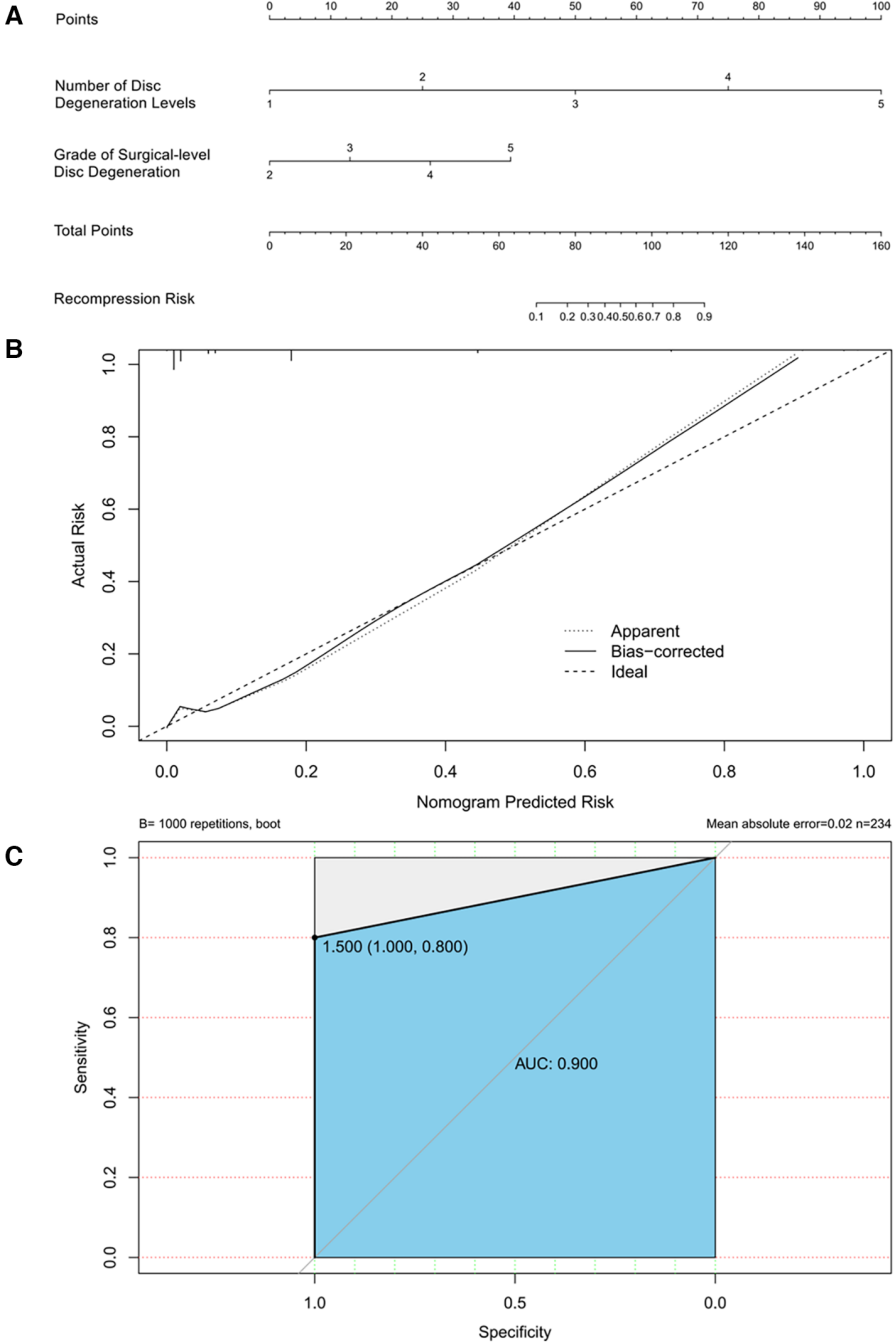
(**A**) Nomogram for predicting recompression after PTED. Points for number of disc degeneration levels and grade of surgical-level disc degeneration can be obtained using a point caliper and then summed to obtain a total score that can be matched to the recompression risk scale. (**B**) Calibration curve of the predictive model. A smaller distance between the bias-corrected curve and ideal curve indicates a better calibration. (**C**) Receiver operating characteristic curve of the predictive model.

A nomogram for predicting recompression after PTED was constructed based on radiological factors selected by logistic regression (Figure 9). A calibration curve of the nomogram indicates that the predicted probability agrees well with the actual recurrence. The area under the receiver operating characteristic curve of this model was 0.900 (95% CI: 0.704–1.000).

Among the 24 patients with recompression, 13 and 11 underwent PTED and PLIF as revision operations, respectively. There was no significant difference in demographic data between the PTED and PLIF groups (Table 5). Similarly, there was no significant difference between the groups per VAS and ODI scores before and after primary PTED. However, patients in the PLIF group had higher VAS of back pain (46.1 vs. 40.0, *p* = 0.001) and leg pain (59.8 vs. 52.2, *p* < 0.001), and higher ODI (54.9 vs. 48.0, *p* < 0.001) before the revision operation.

**Table 5:** Characteristics of patients who underwent PTED and PLIF as reoperation.

	PTED group (*n* = 13)	PLIF group (*n* = 11)	*P* value
Age (years)	69.5 ± 13.6	66.9 ± 9.4	0.606
Gender (male/female)	9/4	7/4	1.000
BMI (kg/m^2^)	24.8 ± 2.1	26.8 ± 4.0	0.160
Surgical level, *n*			0.300
L4/L5	12	8	
L5/S1	1	3	
Time of reoperation (months)	16.4 ± 14.9	20.0 ± 21.5	0.633
Follow-up duration (months)	32.5 ± 17.9	23.6 ± 15.1	0.206

PTED, percutaneous transforaminal endoscopic decompression; PLIF, posterior lumbar interbody fusion; BMI, body mass index.

The post-reoperative VAS for back pain (*p* < 0.001) and leg pain (*p* < 0.001), and ODI (*p* < 0.001) were significantly improved compared with the pre-reoperative values in both groups. Moreover, no significant difference was found in the VAS for back pain (*p* = 0.079), leg pain (*p* = 0.683), and ODI (*p* = 0.463) 2 weeks post-reoperatively between the groups. However, the mean VAS for back pain (14.1 vs. 20.5, *p* = 0.016) and ODI (16.0 vs. 21.8, *p* = 0.016) of patients in the PLIF group were smaller than those in the PTED group at the final follow-up. According to the MacNab criteria, 9 and 10 (69.2% and 90.9%) patients achieved clinical satisfaction in the PTED and PLIF groups, respectively. The results are summarized in Table 6.

**Table 6:** Comparison of clinical outcomes between patients in PTED and PLIF group.

	Overall (*n* = 24)	PTED group (*n* = 13)	PLIF group (*n* = 11)	PTED vs. PLIF
VAS for back pain				
Preoperative	40.5 ± 6.4	40.5 ± 7.4	40.4 ± 5.2	0.948
Two weeks postoperative	16.3 ± 5.0^†^	16.0 ± 4.1^†^	16.7 ± 6.0^†^	0.731
Three months postoperative	17.7 ± 8.9^†^	17.1 ± 7.5^†^	18.3 ± 10.7^†^	0.767
Pre-reoperative	42.8 ± 4.9	40.0 ± 4.3	46.1 ± 3.3	0.001*
Two weeks post-reoperative	16.3 ± 4.5^‡^	17.8 ± 5.6^‡^	14.6 ± 1.9^‡^	0.079
Three months post-reoperative	15.8 ± 4.8^‡^	17.4 ± 5.8^‡^	14.0 ± 2.2^‡^	0.071
One year post-reoperative	17.9 ± 7.1^‡^	20.5 ± 6.4^‡^	14.8 ± 6.9^‡^	0.048*
Final follow-up	17.6 ± 6.8^‡^	20.5 ± 5.9^‡^	14.1 ± 6.3^‡^	0.016*
VAS for leg pain				
Preoperative	55.8 ± 4.9	55.4 ± 4.5	56.1 ± 5.7	0.703
Two weeks postoperative	20.0 ± 6.3^†^	19.2 ± 6.7^†^	21.1 ± 5.9^†^	0.465
Three months postoperative	21.4 ± 11.6^†^	20.2 ± 10.7^†^	22.9 ± 13.0^†^	0.578
Pre-reoperative	55.7 ± 5.4	52.2 ± 3.6	59.8 ± 3.9	<0.001*
Two weeks post-reoperative	20.8 ± 5.0^‡^	21.2 ± 6.2^‡^	20.4 ± 3.4^‡^	0.683
Three months post-reoperative	20.5 ± 5.3^‡^	20.8 ± 6.6^‡^	20.1 ± 3.6^‡^	0.759
One year post-reoperative	21.9 ± 7.0^‡^	23.5 ± 7.8^‡^	20.0 ± 5.7^‡^	0.224
Final follow-up	22.3 ± 7.1^‡^	24.2 ± 7.6^‡^	20.1 ± 5.9^‡^	0.158
ODI				
Preoperative	51.6 ± 4.3	51.8 ± 4.2	51.3 ± 4.6	0.752
Two weeks postoperative	16.8 ± 5.7^†^	16.0 ± 5.9^†^	17.8 ± 5.5^†^	0.447
Three months postoperative	18.1 ± 10.1^†^	16.8 ± 9.7^†^	19.6 ± 10.7^†^	0.499
Pre-reoperative	51.2 ± 5.3	48.0 ± 3.7	54.9 ± 4.3	<0.001*
Two weeks post-reoperative	17.2 ± 3.9^‡^	17.7 ± 5.0^‡^	16.5 ± 2.2^‡^	0.463
Three months post-reoperative	16.5 ± 3.9^‡^	16.9 ± 4.9^‡^	16.0 ± 2.5^‡^	0.577
One year post-reoperative	17.3 ± 5.9^‡^	18.9 ± 6.1^‡^	15.4 ± 5.2^‡^	0.148
Final follow-up	19.2 ± 6.1^‡^	21.8 ± 5.4^‡^	16.0 ± 5.5^‡^	0.016*
Clinical satisfactory, *n* (%)	21 (79.2%)	9 (69.2%)	10 (90.9%)	0.327

* Statistically significant at *p* < 0.05.

^†^ Compared to the preoperative values, results showed a significant difference (paired samples t-test, *p* < 0.001).

^‡^ Compared to the pre-reoperative values, results showed a significant difference (paired samples t-test, *p* < 0.001).

PTED, percutaneous transforaminal endoscopic decompression; PLIF, posterior lumbar interbody fusion; VAS, visual analogue score; ODI, Oswestry disability index.

The complications of reoperations are as follows: Epidural hepatoma occurred in 1 patient after PLIF, and he underwent additional PTED. Persistent lower extremity pain occurred in 1 patient after PLIF and 2 after PTED, and they were all treated by nerve root blocking. Adjacent segment degeneration occurred in 1 patient after PLIF and 1 after PTED. Two patients were treated with additional PTED. There was no significant difference in the prevalence of complications between the two groups (*p* = 1.000).

## Discussion

This study retrospectively reviewed the clinical and imaging data of 820 DLSS patients who underwent PTED at our institution. 26 patients had recompression and underwent a reoperation. Univariate and multivariate logistic regression analyses revealed that the grade of disc degeneration at the surgical level and the number of levels with disc degeneration were associated with recompression after PTED. A Nomogram based on the two radiological parameters can predict recompression after PTED with optimal discrimination and excellent calibration. To determine whether there was a difference between the outcomes of different reoperation procedures due to the imaging characteristics of patients with recompression, we designed a case-control study to compare the outcomes of PTED and PLIF. The final follow-up results showed that the VAS for back pain and the ODI were significantly lower in the PLIF group than in PTED. In addition, there were no significant differences in complications between the two groups. Therefore, we concluded that PLIF may be preferred for the treatment of recompression. However, PTED can act as a practical alternative for patients with advanced age, complex comorbidities, and intolerance to spinal fusion.

The severity of surgical-level disc degeneration is closely related to the clinical manifestations, surgical indications, and postoperative efficacy (28). We considered that recompression was mainly due to severe degeneration of the entire segment. In patients with early recompression, disk is often the cause of radiculopathy. Senior grade disk degeneration indicates a severe annular injury, a fragile structure, and complex adhesions, resulting in a difficulty in annulus repair and a higher risk of nucleus pulposus herniation (29). While in patients with long-term recompression, severe disc degeneration increases the segmental stress force, which leads to the facet joint and ligament flavum hypertrophy and osteophyte formation, resulting in the progression of DLSS (30). However, further pathological evidence is needed to support these conclusions. Adjacent-level degeneration can also increase axial loading (31), which explains why patients with recompression have more degenerated discs. This conclusion is consistent with the previous findings (12, 29). In a recent study, Grieco (32) also reported the association between severe disc degeneration and failure after PTED, attributing it to the potential instability. However, Grieco emphasized that the risk factors are not contraindications for PTED, and instead PTED remains an option due to its better feasibility. This view is consistent with our research.

Older age is related to increased degeneration of the lumbar spine and greater susceptibility to mechanical loading (33), which may explain why age >50 years is considered a risk factor for poor outcome and rLDH postoperatively (13, 34). Geriatric patients often have poor general health. Despite severe lumbar stenosis, they may refuse spinal fusion or be assessed as intolerant to major operations during primary treatment. PTED is less invasive and has lower risks as a substitute; however, these patients may have less satisfactory long-term outcomes and higher recurrence rates (18). In this study, older age was associated with recompression; however, it was not an independent risk factor. We speculated that the probable reason was that some geriatric patients with recompression had relatively poor willingness to undergo treatment and were not evaluated promptly.

Reoperations are often required for patients diagnosed with recompression. The optimal selection of revision surgery was not previously determined. Liu (17) compared the effects and complications of PELD and minimally invasive transforaminal lumbar interbody fusion (MIS-TLIF) to treat rLDH and found that both procedures helped relieve symptoms. However, patients who underwent PELD had a higher postoperative VAS score for back pain and recurrence rate. Similar conclusions were drawn by Yao (18). In this study, we believe that for patients with severe lumbar instability, PLIF is the preferred surgical procedure. Except for this, there is no absolute limit between the indications for PTED and PLIF. We would offer preliminary treatment recommendations to the patients with recompression according to their symptoms and imaging findings. However, the surgical method ultimately needs to be determined in combination with the general condition and willingness of the patients. we found that the surgical outcomes of PLIF were significantly better than those of PTED at the final follow-up. However, Since the comparative analysis was not planned *a priori*, our results are affected by the problems inherent in retrospective analysis. Further prospective randomized controlled trials are required to confirm the findings.

In this study, the results of uni- and multivariate analyses showed that patients with recompression had more severe disc degeneration and more degenerated discs. Based on our experience and the findings of previous studies, we speculate that PLIF would be more effective in such patients compared to PTED. Several theoretical advantages of PLIF are listed as follows: First, multilevel, severe, and bilateral stenosis were often considered indications for spinal fusion in previous studies (21). While PTED can only achieve decompression at the most stenotic level. Second, patients with recompression have more severe degeneration than those without recompression, and the causes of nerve compression are more complex. For these patients, PTED has a limited visual field and operating range. Despite sufficient decompression during surgical exploration, there is still a risk that residual tissue may compress the nerve further. However, PLIF can completely remove the perineural tissues. Osterman (35) found that spinal fusion could reduce the risk for further reoperations. Third, PLIF eliminates segmental motion, stabilizes the spine, and maintains intervertebral and foraminal height (36, 37). By comparison, patients with significant disc collapse and foraminal stenosis may require an excision of more than 50% of the SAP during the revision PTED, which may destroy segmental stability and lead to chronic low back pain (21). However, we found that recompression was also more likely in geriatric patients, so selecting PLIF might lead to several other problems. This method is performed under general anesthesia, which may increase the risk of delayed extubation, delirium, and cognitive dysfunction (38). Owing to osteoporosis and paraspinal muscle degeneration, postoperative pedicle screw loosening and adjacent segment failure are common in geriatric patients (39, 40). Additionally, previous studies have shown that geriatric patients have a poor willingness to revise (41). Therefore, PLIF may be unacceptable in many patients with recompression. Thus, PTED can still be an alternative and shows relatively satisfactory short-term outcomes. Because of further disc degeneration postoperatively, the most common location of recompression is the foramen (14, 23), which provides the theoretical basis for PTED as a surgical choice.

It is noteworthy that regardless of the procedure selected, reoperation is less effective than the primary procedure (42). On the one hand, repeated surgical procedures and further excision of posterior structures increased postoperative back pain (43). On the other hand, distorted anatomical structures and scars due to primary PTED increase the difficulty of revision (44). In addition, releasing adhesive tissue can cause higher rates of dural tearing and epidural hematoma (9, 45). In this study, epidural hematoma occurred in 1 patient, and persistent lower extremity pain occurred in 3 patients. A possible reason could be that meticulous dissection of the nerve root from the adhesions was required during revision, resulting in a higher rate of perineural injury and edema.

This study has several limitations. First, as a retrospective study with a small sample size, it did not allow for entirely precise calculation of true risk or definitive conclusions regarding surgical outcomes. A multicenter prospective study with larger sample size is needed to evaluate the conclusions. Second, due to the limitation of follow-up, some patients with poor compliance might have refused lumbar MRI, which led to a larger number of patients with recompression than we reported. Third, the surgical choices were limited in this study. Other procedures, such as unilateral biportal endoscopy, which may have advantages in central canal decompression, can be discussed in the future.

## Conclusion

This retrospective study indicated that the grade of surgical-level disc degeneration and the number of levels with disc degeneration may be associated with a higher risk for recompression. Although both PTED and PLIF achieved immediate postoperative relief, the efficacy of PLIF appeared better than that of PTED at the final follow-up. Thus, PLIF may achieve relatively better outcomes compared to PTED as revision surgery, given that severe and multilevel degeneration is often considered an indication for spinal fusion. However, PTED could be a viable alternative treatment for patients with advanced age and poor general conditions. Further prospective studies are needed to identify the risk factors and optimal surgical choices for recompression after PTED.

## Data Availability

The raw data supporting the conclusions of this article will be made available by the authors, without undue reservation.
